# Relationship between Daytime Sleepiness and Health Utility in Patients after Cardiac Surgery: A Preliminary Study

**DOI:** 10.3390/ijerph15122716

**Published:** 2018-12-02

**Authors:** Kazuhiro P. Izawa, Yusuke Kasahara, Koji Hiraki, Yasuyuki Hirano, Koichiro Oka, Satoshi Watanabe

**Affiliations:** 1 Department of Public Health, Graduate School of Health Sciences, Kobe University, Kobe 654-0142, Japan; izawapk@ga2.so-net.ne.jp; 2 Department of Rehabilitation Medicine, St. Marianna University School of Medicine Yokohama-City Seibu Hospital, Yokohama 241-0811, Japan; kasahara.y@marianna-u.ac.jp; 3 Department of Rehabilitation Medicine, St. Marianna University School of Medicine Hospital, Kawasaki 216-8511, Japan; hiraki7@marianna-u.ac.jp; 4 Department of Physical Therapy, Tokushima Bunri University, Tokushima 770-8514, Japan; hirano@tks.bunri-u.ac.jp; 5 Faculty of Sport Sciences, Waseda University, Tokorozawa 359-1192, Japan; koka@waseda.jp; 6 Cardiovascular stroke Renal Project (CRP), Kobe 654-0142, Japan

**Keywords:** age, Short-Form Six-Dimension, Epworth Sleepiness Scale, cardiac surgery

## Abstract

*Background* Daytime sleepiness can be assessed by the Epworth Sleepiness Scale (ESS), which is widely used in the field of sleep medicine as a subjective measure of a patient’s sleepiness. Also, health utility assessed by the mean Short-Form Six-Dimension (SF-6D) score, one of several preference-based utility measures, is an important measure in health care. We aimed to examine age-related differences in daytime sleepiness and health utility and their relationship in patients 5 months after cardiac surgery. *Methods*; This cross-sectional study assessed 51 consecutive cardiac surgery patients who were divided into a middle-aged (<65 years, *n* = 29) and older-age group (≥65 years, *n* = 22). The mean ESS and SF-6D utility scores were measured at 5 months after cardiac surgery and compared. In addition, the relationship between ESS and SF-6D utility scores were assessed. *Results*; There were no significant differences between the middle-aged and older-aged groups in either the mean ESS (5.14 ± 2.96 vs. 4.05 ± 3.23, *p* = 0.22) or SF-6D utility (0.72 ± 0.14 vs. 0.71 ± 0.10, *p* = 0.76) scores. However, there was a negative correlation between both values in all of the patients after cardiac surgery (*r* = −0.41, *p* = 0.003). *Conclusions*; Although there were no age-related differences in the ESS and SF-6D utility values between the two groups, there was a negative correlation between these values in all patients at 5 months after cardiac surgery. This suggested that sleepiness is associated with decreased utility scores in patients at 5 months after cardiac surgery.

## 1. Introduction

Poor sleep quality, sleeplessness, and sleep disorder are common among patients recovering from cardiac surgery such as coronary artery bypass grafting (CABG) [1,2]. Previous studies have suggested that they may have important effects on morbidity and mortality [1,2,3,4]. Moreover, patients with cardiac surgery often report sleeping difficulties (e.g., initiating, maintaining sleep, and daytime sleepiness) that are associated with decreased quality of life (QOL) [1,3]. In addition, several studies reported that sleep is disturbed in the early period after surgery [3,4,5]. However, there is little reported evidence on sleep disturbance in the chronic phase after cardiac surgery. Sleep disorder in cardiac patients can be diagnosed using polysomnography, heart rate variability, and questionnaires [6], which have recently been effective in the screening of sleep apnea. Because of the limited accessibility and cost of polysomnography, questionnaires have been advocated to identify patients who are likely to have a sleep disorder. These questionnaires can help to assess the extent of sleepiness or wakefulness. Other tools with which to assess sleep disorder include subjective measures such as the Epworth Sleepiness Scale (ESS) [7] in addition to an objective measure such as polysomnography [6,8]. Excessive sleepiness may have effects on education, employment, and interpersonal relations, and it can directly degrade a person’s QOL, especially relative to health, functional capacity, and sense of well-being [9,10]. We previously suggested that sleep disorder in cardiac patients such as those with chronic heart failure may cause sleepiness that presumably can lead to a reduction in QOL [11].

Cardiac rehabilitation (CR) in cardiac patients helps to reduce risk factors, improve exercise capacity and health-related QOL, lower the number of subsequent cardiac events, and reduce all-cause mortality and hospitalization costs [12,13,14,15,16]. In terms of these costs, several previous studies suggested that health utility, which is assessable by several preference-based utility measures, is an important measure when analyzing cost effectiveness in health care [17,18,19,20]. The Short-Form Six-Dimension (SF-6D), an instrument that provides a summary score based on the Short Form-36 (SF-36), was recently developed [17,18,19,20] to enlarge the bases for economic evaluations while retaining the descriptive richness and sensitivity to change of the SF-36 [17,18,19,20]. Previously, we tried to identify and compare age-related differences in health utility assessed by mean SF-6D utility score in 62 cardiac surgery patients at 1 and 3 months after cardiac surgery [21]. However, we could find no evidence of a relation between age-related differences and health utility assessed by the SF-6D in the chronic phase following cardiac surgery. Furthermore, there is very little evidence on the effects of age-related differences on sleep quality and health utility in patients in the chronic phase after cardiac surgery.

Therefore, we hypothesized that there might be age-related differences in daytime sleepiness and health utility, and daytime sleepiness might be related to health utility as evaluated by self-reported questionnaire. The purpose of the present study was to determine both age-related differences in daytime sleepiness and health utility and the relationship between daytime sleepiness and health utility in patients at 5 months after cardiac surgery as assessed by self-reported questionnaires.

## 2. Materials and Methods

This cross-sectional study comprised 67 consecutive cardiac patients with coronary artery bypass grafting or valve replacement and valvuloplasty who visited hospital as outpatients 5 months after cardiac surgery and were referred to the department of rehabilitation medicine for the evaluation of daytime sleepiness and health utility. The patients were initially divided into a middle-aged group (<65 years, *n* = 36) and older-age group (≥65 years, *n* = 31). We reviewed the patients’ medical records to evaluate the following patient characteristics of age, sex, body mass index (BMI), etiology, left ventricular ejection fraction (LVEF), and medications. These characteristics and daytime sleepiness and health utility were assessed at 5 months after cardiac surgery and compared between the two groups. Exclusion criteria included patients with neurological, peripheral vascular, orthopedic, pulmonary, and advanced renal disease and those on dialysis. The present study complied with the principles of the Declaration of Helsinki regarding human investigations and was approved by the local institutional review board. All patients provided their informed consent.

### 2.1. ESS as an Index of Daytime Sleepiness

We evaluated self-reported daytime sleepiness via questionnaire by using the Japanese version of the ESS [22,23]. The ESS, which is widely used in many countries to investigate daytime sleepiness, measures the tendency to sleep or doze during active and passive situations commonly encountered while awake. The ESS comprises eight questions. By summing the scores (ranging from 0–3) for each question, total scores of 0–24 are calculated. A higher total score indicates increased severity of daytime sleepiness, with ESS scores of 11 or higher signifying excessive daytime sleepiness. After evaluating the sleepiness of the study patients, we calculated their average ESS scores.

### 2.2. SF-6D as an Index of Health Utility

Health utility was measured using mean SF-6D utility scores. The SF-6D was developed as a practical tool to obtain a preference-based index from SF-36 data [24,25]. First, we assessed the patients with the SF-36. The SF-6D questionnaire was developed to obtain health utility from the SF-36 questionnaire for use in health economic evaluations and links between psychometric and preference/utility-based measures [24,25]. If there are no limitations in any of the dimensions, no value is subtracted from the baseline value of 1.0, which indicates perfect health [24,25]. The higher the limitation in each domain, the higher the subtraction from the baseline. After we assessed the patients with the SF-36, the scores were converted to mean SF-6D utility scores by iHope International Co. Ltd. (Kyoto, Japan) based on techniques used in previous reports [17,19,20,21].

### 2.3. Statistical Analysis

Results are expressed as the mean ± standard deviation (SD). We used the unpaired *t*-test and χ^2^ test to test for differences between the middle-age and older-age groups in clinical characteristics and ESS and mean SF-6D utility scores. We also analyzed the relationship between the ESS and SF-6D utility scores using the Pearson correlation coefficient. A *p* value of < 0.05 was considered to indicate statistical significance. Statistical analyses were performed with IBM SPSS 23.0 J statistical software (IBM SPSS Japan, Inc., Tokyo, Japan).

## 3. Results

### 3.1. Clinical Characteristics of the Patients

Of the 67 patients, 16 were excluded from the study because of lack of data on clinical characteristics, the ESS, and the SF-6D utility score at 5 months after cardiac surgery. Thus, of the remaining 51 patients, 29 comprised the middle-aged group, and 22 comprised the older-age group. Also, it appeared that both groups experienced practically no decompensations during the initial 5 months post discharge. Except for age, the clinical characteristics were not significantly different between the two groups (Table 1).

### 3.2. Sleep Quality and Health Utility at 5 Months after Cardiac Surgery

**Figure 1:**
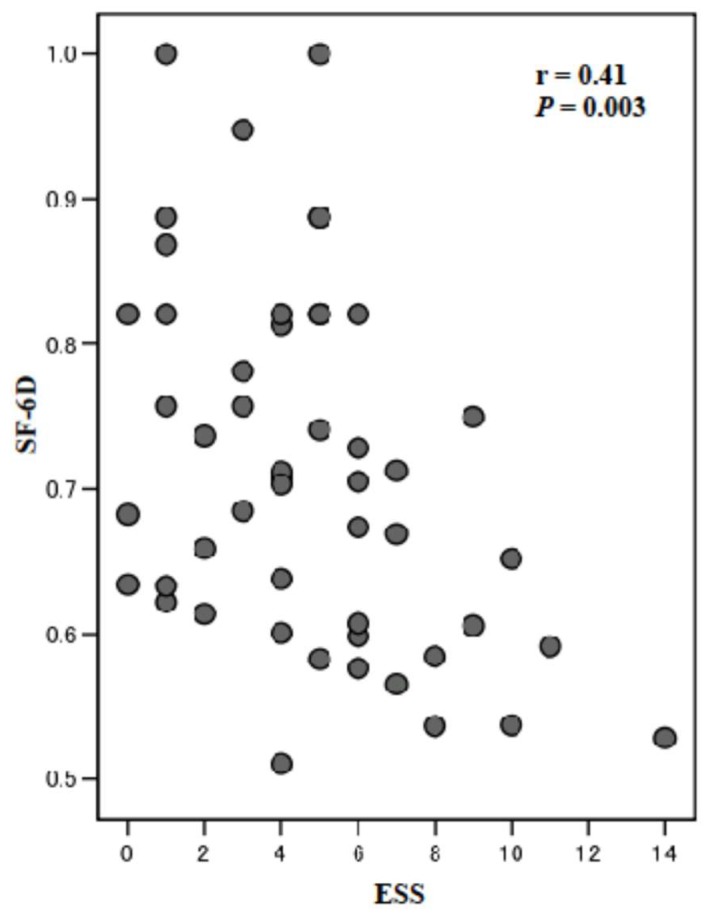
Relationship between daytime sleepiness and health utility in the study patients at 5 months after cardiac surgery. There was a significant negative correlation between mean ESS score and the SF-6D utility score in the patients. ESS, Epworth Sleepiness Scale; SF-6D, Short-Form Six-Dimension.

The mean ESS and SF-6D utility scores collected from the two groups are presented in Table 2. There were no significant differences in these scores between the middle-aged group and older-aged group at 5 months after cardiac surgery. However, as shown in Figure 1, there was a statistically significant negative correlation between the mean ESS and SF-6D utility scores for all of the patients at 5 months after cardiac surgery (*r* = −4.10, *p* = 0.003).

## 4. Discussion

To our knowledge, this is first time that the mean ESS as an index of daytime sleepiness and the mean SF-6D utility score as an index of health utility have been evaluated in relation to age in cardiac surgery patients at 5 month after surgery. Moreover, the relationship between these two values was also evaluated. The main finding of this study was that there were no significant differences in the mean ESS and SF-6D utility score values at 5 months after cardiac surgery between the middle-aged and the older-aged groups. However, there was a negative correlation between two these values in all patients.

With the exception of age, all other clinical characteristics of the patients were almost identical between the middle- and older-aged groups. Therefore, clinical characteristics of the patients other than age likely had little effect on any differences in the mean ESS and SF-6D utility scores found in the two groups. We evaluated daytime sleepiness status with the ESS because it is well validated, and scores from several subject groups have been published [7,8,23,24]. Because the ESS scores in our study were not significantly different between the two groups, we thus thought that the ESS as an indicator of the degree of daytime sleepiness reflected the status of the middle aged cardiac patients equally as well as that of the older-age patients. Previous reports have focused on an age bias in the approach to treating elderly cardiac patients [26,27,28]. However, a recent study reported that although their results did not differ significantly based on age, sex, emergent status, or history of heart failure or arrhythmias, participation in CR was of more benefit to the patients who underwent cardiac surgery [29]. In addition, we previously reported that no age-related differences in health utility were found between middle-aged and older patients upon entrance into a phase II CR program at 1 month and at 3 months after cardiac surgery [21]. This indicates that aging might not reflect differences in the average SF-6D utility scores of patients at 5 months after cardiac surgery, and these findings might support those of the previous study [21].

In a cost-utility analysis carried out in tandem with a randomized controlled trial using the SF-6D in learning and coping strategies in CR that comprised 825 patients over 18 years of age admitted with ischemic heart disease or heart failure, Dehbarez et al. reported mean SF-6D scores of 0.739 at baseline, 0.798 at 2 months after intervention, and 0.794 after 5 months of follow-up [30]. Although there were differences between etiologies and CR programs between the Dehbarez et al. study [30] and the present study, the respective mean SF-6D utility scores in the middle and older-aged groups at 5 months in the present study were 0.72 and 0.71, which were lower than those of the cardiac patients studied by Dehbarez et al. Therefore, we need to evaluate whether health utility continues to change over the long term following a phase II CR outpatient program. The negative correlation between the mean ESS and SF-6D utility scores at 5 months after cardiac surgery in the present study was moderate (*r* = 0.41, with *r* < 0.4 indicating a weak, *r* ≥ 0.4 but <0.6 indicating a moderate, and *r* ≥ 0.6 but <0.8 a strong correlation [31]). Lopes et al. [10] showed that excessive sleepiness and lack of physical activity affect the QOL of apneic patients, which was worse among their sleepy non-physically active subjects and increasingly worse in their patients with severe apnea. In the present study, the mean ESS scores in the middle and older-aged groups at 5 months were 5.14 and 4.05, respectively (Table 2).

ESS scores of 11 or higher indicate excessive daytime sleepiness. In some reports, cardiac patients such as those who underwent CABG complained of sleeplessness [32], and sleep disturbance persisted for weeks, months, or nearly 1 year after heart surgery [33,34,35]. One study suggested that sleep improved at 6 months [36]. The mean ESS scores at 5 months of our middle (5.14) and older-aged (4.05) patients were less than 11, indicating that sleepiness may have improved at 5 months after cardiac surgery. However, we did not investigate whether the ESS scores improved from early after discharge from hospital to 5 months later. Moreover, shallow sleep or poor sleep quality may cause sleepiness, and presumably, increased sleepiness may be related to a reduction in health utility. Therefore, there is a need for further studies to confirm our findings in a larger number of patients to determine longitudinally whether daytime sleepiness improves after cardiac surgery and to ascertain a causal relationship between daytime sleepiness and health utility.

There are several limitations in the present study. The sample size is very small and is comprised almost entirely of male cardiac surgery patients. Additional analysis of sex-related differences in daytime sleepiness and health utility in female cardiac patients is also required. We did not evaluate the effects of postoperative complications or the start day of an earlier in-hospital rehabilitation program on selection bias, or of longer hospital stays. We also did not examine the presence or absence of drugs and patient age in relation to daytime sleepiness.

In addition, the analyses reported in Table 1 and Table 2 reveal that patients’ normal weight, and/or BMI reinforces the data found in relation to ESS, as the low and/or high specificity may be related to the younger and/or older age of the patients. This may be suggestive of a biased sample population. Moreover, in the inclusion criteria, we did not differentiate between the type of CABG the patients underwent, and this may reinforce differences in the quality of health and sleep in accordance with the complexity of the different types of surgery undergone. There was no analysis and no correlation of benefit between those who did or did not participate in the CR program after cardiac surgery. In our future research, we need to increase both the number of patients studied and the follow-up time analyzed as the types of complications occurring in the postoperative period following cardiac surgery take place over the long term.

A recent study reported that cardiac surgery is correlated with obstructive sleep apnea (OSA) in cardiovascular outcomes after a period of 4.5 years, where it was shown that OSA is independently associated with a higher rate of long-term cardiovascular events after CABG and may have prognostic and economic significance in CABG surgery [37]. We did not examine OSA in relation to daytime sleepiness in the present study. In addition, there are no data on sleep problems experienced before cardiac surgery. The lack of socioeconomic data [38] may have also affected study results. Thus, we need to address these deficiencies in future longitudinal studies.

## 5. Conclusions

This study attempted to identify both age-related differences in sleep quality and health utility and the relationship between these values in patients in the chronic phase at 5 months after cardiac surgery. We found no significant age-related differences between sleep quality and health utility at 5 months after cardiac surgery. However, there was a significant negative correlation between these values in all patients. Because this study lacks long-term follow-up data on sleep quality and health utility, additional study will be required to evaluate outcomes over longer periods of time in these patients.

## Figures and Tables

**Table 1:** Clinical characteristics of the patients.

Clinical Characteristics	Middle-Aged Group	Older-Aged Group	*t* or χ^2^ Value *	*p* Value
No. of patients	29	22		
Age (yrs)	56.3 ± 7.1	70.9 ± 3.6	−8.61	<0.001
Sex (male)	23	18	0.02 *	0.58
BMI (kg/m^2^)	23.4 ± 2.4	22.7 ± 3.0	0.88	0.38
LVEF (%)	52.8 ± 11.0	57.3 ± 12.1	−1.04	0.31
Etiology (%)				
CABG	62.1	71.4	0.47 *	0.35
VR/VP	37.9	28.6	-	-
Medications (%)				
Beta-blockers	43.7	45.5	0.00 *	0.61
ACEI/ARB	31.2	54.5	1.46 *	0.20
Diuretic	62.5	81.8	2.73 *	0.11

BMI body mass index, LVEF left ventricular ejection fraction, CABG coronary artery bypass grafting, VR valve replacement, VP valvuloplasty, ACEI, angiotensin converting enzyme inhibitor; ARB, angiotensin receptor blocker. * χ^2^ value.

**Table 2:** Differences in ESS and SF-6D scores at 5 months after cardiac surgery.

	Middle-Aged Group	Older-Aged Group	*t* Value	*p* Value
ESS	5.14 ± 2.96	4.05 ± 3.23	1.24	0.22
SF-6D	0.72 ± 0.14	0.71 ± 0.10	0.31	0.76

ESS Epworth Sleepiness Scale, SF-6D Short-Form Six-Dimension.
